# Immunophenotypic, Genetic, and Clinical Features Associated With *RUNX1* Mutation in Acute Leukemias and Chronic Myeloid Neoplasms

**DOI:** 10.1111/ijlh.70145

**Published:** 2026-05-15

**Authors:** Yi Han Xia, Eric McGinnis

**Affiliations:** ^1^ Faculty of Medicine University of British Columbia Vancouver British Columbia Canada; ^2^ Vancouver General Hospital Vancouver British Columbia Canada; ^3^ Department of Pathology and Laboratory Medicine University of British Columbia Vancouver British Columbia Canada

**Keywords:** acute, antigens, B‐lymphocyte, differentiation, flow cytometry, leukemia, myelodysplastic syndromes, myeloid, runt‐related transcription factor 1

## Abstract

**Introduction:**

RUNX1 is a commonly mutated transcriptional regulator of hematopoiesis in acute myeloid leukemia (AML) and myelodysplastic syndrome (MDS). Mutated *RUNX1* (m*RUNX1*) may associate with cross‐lineage immunophenotypic aberrancy, presenting potential complications for blast lineage assignment at diagnosis.

**Methods:**

Clinical and laboratory data were reviewed for patients with *RUNX1* mutations (*N* = 125) at Vancouver General Hospital from 2016 to 2022. Diagnostic flow cytometry data were reanalyzed, and *RUNX1* mutation characteristics and associations with pathologic, comutation, and flow cytometric features were assessed.

**Results:**

Missense *RUNX1* mutations (overall 42.9% missense, 57.1% truncating) were enriched in the runt homology domain (RHD; 81.7%, Adj. *p* = 0.011). Several recurrent hotspot mutations were seen, including common hotspot variants. The most commonly comutated genes with *RUNX1* in both acute leukemia with m*RUNX1* and MDS‐m*RUNX1* were *ASXL1* (44.4%; 59.3%), *SRSF2* (40.7%; 37.3%), and *TET2* (35.8%; 55.9%). The m*RUNX1* group demonstrated frequent positivity for B‐lymphoid markers, with 14.4% having increased expression in at least three B‐lineage markers. Differences in antigen expression were associated with *RUNX1* mutation type and location, with increased CD10 expression associated with *RUNX1* mutations in the RHD (Adj. *p* = 0.02764), and increased CD79a expression associated with *RUNX1* missense mutations (Adj. *p* = 0.004452).

**Conclusion:**

In our cohort, we observed missense variants clustering in the RHD and recurrence of common pathogenic variants. Higher‐risk comutations were found in a substantial fraction of m*RUNX1* cases which may contribute to its adverse risk associations. Comparable degrees of aberrancy in B‐lineage markers were seen in m*RUNX1* patients versus the comparator cohorts of MPAL‐wt*RUNX1* and AML with rearranged *RUNX1*.

## 1 Introduction

Runt‐related transcription factor 1 (*RUNX1*), also known as acute myeloid leukemia 1 protein (*AML1*), is a critical transcription factor in the regulation of hematopoietic stem cell differentiation and proliferation [1, 2]. *RUNX1* encodes the DNA‐binding subunit of core‐binding‐factor alpha, interacting with DNA primarily through the runt homology domain (RHD) [3, 4]. Other domains of *RUNX1* include the transactivation domain (TAD) and the runt inhibitory domain (RID) that interact with other proteins to regulate the expression of target genes [1]. Initially discovered as the fusion partner of *RUNX1T1* (*ETO*) in acute myeloid leukemia (AML) with *t*(8;21)(q22;q22)/*RUNX1::RUNX1T1*, *RUNX1* mutations have been found to be relatively common in AML and chronic myeloid neoplasms such as myelodysplastic syndrome (MDS) and MDS/myeloproliferative neoplasm (MDS/MPN) overlap syndromes, in which *RUNX1* mutations have been shown to occur in about 10% of AML and MDS cases [1, 5, 6, 7]. Pathogenic *RUNX1* variants in the germline context are associated with inherited thrombocytopenia and predisposition to hematologic (primarily myeloid) malignancy [8].

In AML with normal karyotype, the presence of mutated *RUNX1* (m*RUNX1*) has been previously shown to be associated with a poorer prognosis compared to wild type *RUNX1* [9]. The pathologic significance of *RUNX1* mutations in AML was recognized in the World Health Organization (WHO) 4th edition classification of hematologic malignancies, where AML with m*RUNX1* was designated as a provisional diagnostic entity [10]; however, this provisional entity was removed in the most recent edition due to insufficient evidence defining it as a distinct pathologic entity [11]. In contrast, *RUNX1* mutations were retained in the International Consensus Classification (ICC) of Myeloid Neoplasms and Acute Leukemias as one of the myelodysplasia‐related gene mutations categorizing AML and MDS [12]. The exclusion of AML with m*RUNX1* from the latest WHO classification while being preserved in the ICC underscores the continuously increasing complexity of AML classification in the context of a deepening understanding of the molecular underpinnings of myeloid neoplasms and highlights the need for more precise biomarkers to guide diagnosis and therapy.

Immunophenotypic aberrancy in the form of cross‐lineage B‐lymphoid antigen expression is classically associated with *RUNX1* rearrangements in AML (AML‐r*RUNX1*), which have demonstrated gene expression profiles featuring enhanced activity in pathways associated with lymphoid differentiation [9, 13]. *RUNX1* mutations have been associated with similar phenomena—for example, aberrant cross‐lineage antigen expression has been reported in AML with m*RUNX1*, including expression of typical B‐lineage antigens such as PAX5, CD19, CD20, CD22, and CD79a [14, 15]. Aberrant cross‐lineage antigen expression can complicate lineage assignment in acute leukemias, and thereby in some instances appropriate treatment selection; variable aberrancy observed in some instances may blur diagnostic lines with mixed phenotype acute leukemia (MPAL) [14, 16].

In the context of the evolving diagnostic role of *RUNX1* mutations in myeloid neoplasms and their potential to influence immunophenotype and lineage‐based classification, we evaluated a cohort of patients with *RUNX1*‐mutated myeloid neoplasms for cytogenetic, molecular, immunophenotypic, and clinicopathologic associations with their mutational profile.

## 2 Materials and Methods

### 2.1 Study Cohort and Clinical Data Collection

We retrospectively reviewed all identified *RUNX1*‐mutated myeloid malignancies with diagnostic flow cytometry studies performed at Vancouver General Hospital in the seven‐year period following deployment of clinical next‐generation sequencing (NGS) ‐ between January 2016 and December 2022. Chart review was conducted using electronic medical records for demographic data, laboratory data including hemoglobin, neutrophil, platelet, and blast counts at diagnosis, cytogenetic abnormalities, bone marrow morphologic findings, occurrence of relapse or death, and occurrence of hematopoietic stem cell transplantation (HSCT). Outcomes were censored to the end of January 1, 2025. Patients were identified for inclusion by laboratory database query for NGS reports including a reported pathogenic *RUNX1* mutation. Comparator groups of patients with AML‐r*RUNX1* and MPAL with wild type *RUNX1* (MPAL‐wt*RUNX1*) were assembled through query of local pathology records. This study was approved by the UBC Clinical Research Ethics Board (H23‐01478).

### 2.2 Diagnostic Flow Cytometry Analysis

Standard diagnostic flow cytometry data were reanalyzed using FlowJo v10. Doublets were initially excluded using forward scatter height and forward scatter area. Myeloid blast populations were identified by combinations of light scatter‐based gating and expression of CD45, CD34, CD117, and/or other features of myeloid precursor differentiation. Lymphocytes were also identified by gating with markers of B‐lymphoid or T‐lymphoid differentiation. Median fluorescence intensity (MFI) was captured for markers commonly associated with B‐lymphoid differentiation (CD10, CD19, CD20, CD22, CD79a), common aberrant cross‐lineage antigens (CD7, CD56), a key antigen defining T‐lymphoid differentiation (CD3), and antigens associated with myeloid differentiation (MPO) or immaturity (CD117, CD34, CD133, TdT). When available, these were captured for blast populations, B‐lymphocytes, T‐lymphocytes, and lymphocytes overall. Relative fluorescence intensity (RFI) was calculated as a ratio of blast MFI to the mean negative control MFI of each marker. The negative controls used were T‐lymphoid expression for B‐lymphoid markers and vice versa, and mature lymphocytes for markers associated with myeloid differentiation or immaturity. The RFI for samples with a blast MFI that was less than the mean negative control MFI was adjusted to be equal to one. Marker expression was also assessed as a binary parameter with positivity for increased expression of each marker defined as having greater than 80% (determined after review of data that corresponded with reasonable lymphocyte marker positivity rates in the comparator cohorts) of the blast population having a fluorescence greater than the MFI of the respective negative control.

### 2.3 Statistical Analysis

Clinical, laboratory, and demographic features were compared between groups, and patterns of antigenic aberrancy were evaluated for association with key elements of these features. Descriptive statistics for demographics, presenting clinical features, data regarding clinical course, and laboratory parameters were prepared (Table 1). Categorical variables were compared using the Fisher exact test. Continuous variables were compared using two‐sample *t*‐test or Wilcoxon signed‐rank test, depending on the normality of data. Pearson correlation compared different continuous variables to each other. Analysis of variance with Tukey's test or Kruskal‐Wallis test with Dunn's test assessed numerical variables among comparisons between three or more groups, depending on normality. Overall survival (OS) was calculated from the diagnosis date to the date of death or censored to the date of most recent documented follow‐up using the Kaplan–Meier method. Multivariate Cox regression adjusted for diagnostic group, age at diagnosis, sex, and cytogenetic complexity (three or more cytogenetic abnormalities) when possible. Statistical analyses were performed with a threshold for statistical significance of two‐sided *p* < 0.05, and Benjamini‐Hochberg adjustment for multiple hypothesis testing was applied where applicable. All statistical analyses were performed using R version 4.4.1 [17]. Other R packages used include “tidyverse” for analysis and visualization and “survival” for survival analysis [18, 19].

**TABLE 1:** Demographic and clinical parameters compared between acute leukemia with mutated *RUNX1* and MDS and MDS/MPN with mutated *RUNX1*.

Clinical parameters	Total (*N* = 125)	Acute leukemia with m*RUNX1* (*N* = 70)	MDS and MDS/MPN with m*RUNX1* (*N* = 55)	*p*
Age at diagnosis, years				0.5776
Median (range)	67 (18–93)	69 (28–85)	69 (48–85)	
Sex, *n* (%)				0.1335
Male	82 (65.6)	50 (28.6)	32 (58.2)	
Female	43 (34.4)	20 (71.4)	23 (41.8)	
Overall survival, months				0.1772
Median (95% CI)	18.3 (11.9–26.0)	9.1 (6.2–26.0)	22.4 (17.9–31.0)	
Initial diagnosis, *n* (%)				
AML	67 (50.4)	67 (95.7)		
MDS	43 (34.4)		43 (78.1)	
MDS/MPN	12 (9.6)		12 (21.8)	
MPAL	3 (2.4)	3 (4.3)		
AML subtype, *n* (% of AML)				
*CEBPA*		1 (1.5)		
*MECOM*		1 (1.5)		
MR		56 (83.6)		
*NPM1*		2 (3.0)		
pCT		3 (4.5)		
AML, NOS		4 (6.0)		
MPAL subtype, *n* (% of MPAL with m*RUNX1*)				
*BCR*::*ABL1*		1 (33.3)		
B/myeloid		2 (66.7)		
MDS or MDS/MPN Subtype, *n* (% of MDS)				
IB1			12 (21.8)	
IB2			18 (32.7)	
LB‐MLD			6 (10.9)	
LB‐SLD			4 (7.3)	
pCT			1 (1.8)	
*SF3B1*			2 (3.6)	
CMML‐1/CMML‐2			12 (21.8)	
Karyotypic abnormalities, *n* (%)				
+8	13 (10.4)	9 (12.9)	4 (7.3)	
−7	6 (4.8)	4 (5.7)	2 (3.6)	
del(20q)	5 (4.0)	2 (2.9)	3 (5.5)	
+13	5 (4.0)	5 (7.1)	0 (0)	
del(5q)	5 (4.0)	4 (5.7)	1 (1.8)	
+21	4 (3.2)	3 (4.3)	1 (1.8)	
+11	4 (3.2)	4 (5.7)	0 (0)	
*t*(3q)	4 (3.2)	3 (4.3)	1 (1.8)	

Abbreviations: AML, acute myeloid leukemia; CMML, chronic myelomonocytic leukemia; IB, increased blasts; LB, low blasts; MDS, myelodysplastic syndrome; MLD, multilineage dysplasia; MPAL, mixed phenotype acute leukemia; MPN, myeloproliferative neoplasm; MR, myelodysplasia‐related; pCT, postcytotoxic therapy; SLD, single lineage dysplasia.

## 3 Results

### 3.1 Study Cohort Presenting Parameters and Outcomes

A total of 125 patients were included, ranging in age at diagnosis from 18 to 93 years with a median of 67 years (interquartile range [IQR]: 57–74 years) (Table 1). The cohort was predominantly male (66%). There were 70 patients (44.0%) in the acute leukemia with m*RUNX1* (AL‐m*RUNX1*) group and 55 patients (34.6%) in the MDS & MDS/MPN with m*RUNX1* group (MDS‐m*RUNX1*), with the comparator groups comprising 19 patients (12.0%) in the AML‐r*RUNX1* group and 15 patients (9.4%) in the MPAL‐wt*RUNX1* group. Within the MPAL‐wt*RUNX1* group, the subtypes included four B/myeloid, seven T/myeloid, two B/T, and two *BCR::ABL1* positive cases. Among the subtypes of AML as defined by the WHO 5th edition classification, myelodysplasia‐related AML was the most common (65.1%). Patients receiving HSCT were in the minority (27.7%) overall and within the AL‐m*RUNX1* (34.3%) and MDS‐m*RUNX1* (18.2%) groups. Remission was achieved in a minority of the AML patients (25.6%). A small number of patients had complex karyotypes, with 4 patients (5.7%) in the AML‐r*RUNX1* group and 1 patient (1.8%) in the MDS‐m*RUNX1* group.

### 3.2 Characteristics of *RUNX1* Mutations and Comutations

**FIGURE 1:**
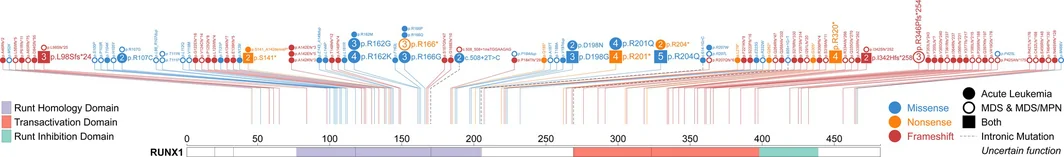
Missense *RUNX1* mutations (blue) appeared more often in the runt homology domain (RHD) and less often in the transactivation domain. Frameshift (red) and nonsense (orange) mutations more commonly clustered downstream of the RHD in the C‐terminus. Recurrent mutational hotspots include R162, R166, D198, and R204.

Among the patients with *RUNX1* mutations, 42.9% had missense *RUNX1* mutations (AL‐m*RUNX1*: 44.5%; MDS‐m*RUNX1*: 40.7%), 14.3% had nonsense mutations (AL‐m*RUNX1*: 13.6%; MDS‐m*RUNX1*: 15.2%), and 42.9% had frameshift mutations (AL‐m*RUNX1*: 42.0%; MDS‐m*RUNX1*: 44.0%). Significant associations were found between *RUNX1* mutation type and distribution in the RHD, TAD, RID, and undefined regions (*p* < 0.001). Missense *RUNX1* mutations were enriched in the RHD compared to non‐RHD regions (81.7%, Adj. *p* = 0.01058; AL‐m*RUNX1*: 86.1%, Adj. *p* = 0.2213; MDS‐m*RUNX1*: 75.0%, Adj. *p* = 1.0) and were sparse in the TAD (0% overall) (Figure 1). Several recurrent hotspot mutations were observed, including 8 missense mutations affecting residue R162 (all of which had AL‐m*RUNX1* diagnoses); 5 each affecting residues R166, D198, and R204; and 4 affecting residue R201. Mean variant allele frequency (VAF) was 33.4% (range: 5.8%–95.7%), which did not vary significantly between diagnoses, mutation regions, or mutation types. The proportion of *RUNX1* mutations with a VAF > 40% was 42.1%.

**FIGURE 2:**
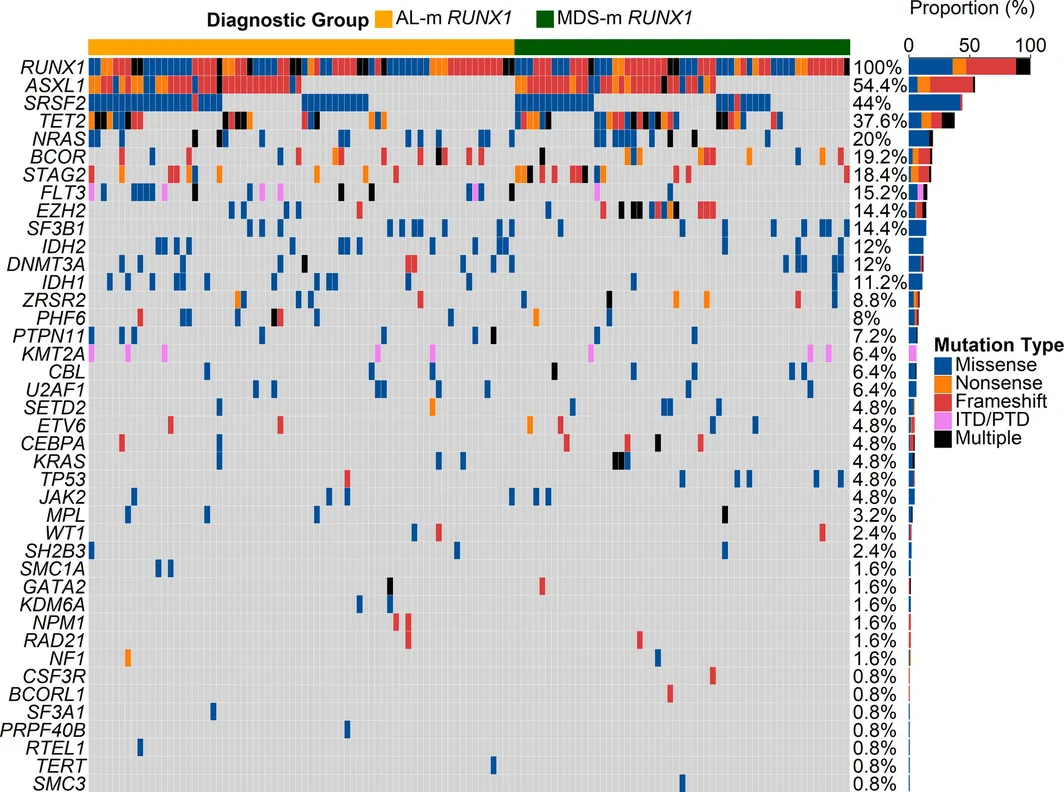
The co‐mutational profile of *RUNX1* was similar between the groups of acute leukemia with mutated *RUNX1* (AL‐m*RUNX1*) and MDS and MDS/MPN with mutated *RUNX1* (MDS‐m*RUNX1*), with genes that were most frequently co‐mutated being *ASXL1*, *SRSF2*, and *TET2*. ITD/PTD: internal tandem duplication/partial tandem duplication.

Common comutations of *RUNX1* were *ASXL1* (44.4% of AL‐m*RUNX1*; 59.3% of MDS‐m*RUNX1*), *SRSF2* (40.7%; 37.3%), and *TET2* (35.8%; 55.9%) (Figure 2). Diagnosis was significantly associated with *FLT3* comutation, in which the AL‐m*RUNX1* group was more likely to have comutated *FLT3* (OR = 6.53, Adj. *p* = 0.04778). The number of comutations did not significantly differ by diagnosis (*p* = 0.7348).

### 3.3 Relationship of Clinical and Genetic Features

Comparing *RUNX1* mutation characteristics with clinical features, platelet count differences were observed in certain groups. In the overall cohort, mean platelet count among *RUNX1* mutations in the undefined region (*M* = 183.16 × 10^12^/L, SD = 159.62 × 10^12^/L) was generally found to be higher than that in the other defined regions (RHD: *M* = 98.26 × 10^12^/L, SD = 100.40 × 10^12^/L, Adj. *p* = 0.004997; RID: *M* = 69.40 × 10^12^/L, SD = 38.15 × 10^12^/L, Adj. *p* = 0.1514; TAD: *M* = 78.00 × 10^12^/L, SD = 85.55 × 10^12^/L, Adj. *p* = 0.003248). Similar differences were observed when divided between the MDS‐m*RUNX1* and AL‐m*RUNX1* groups that trended towards significance.

The number of comutations was significantly associated with shorter OS overall (HR = 1.06, 95% CI = [1.03, 1.09], *p* < 0.001) and within the MDS‐m*RUNX1* (HR = 1.07, 95% CI = [1.03, 1.12], Adj. *p* = 0.00158) and AL‐m*RUNX1* groups (HR = 1.06, 95% CI = [1.02, 1.09], Adj. *p* = 0.00158). Cytogenetic complexity was also associated with shorter OS overall (HR = 2.88, 95% CI = [1.34, 6.20], *p* = 0.00657).

### 3.4 Relationships of Immunophenotypic Expression and Clinical Parameters and Genetic Features

**FIGURE 3:**
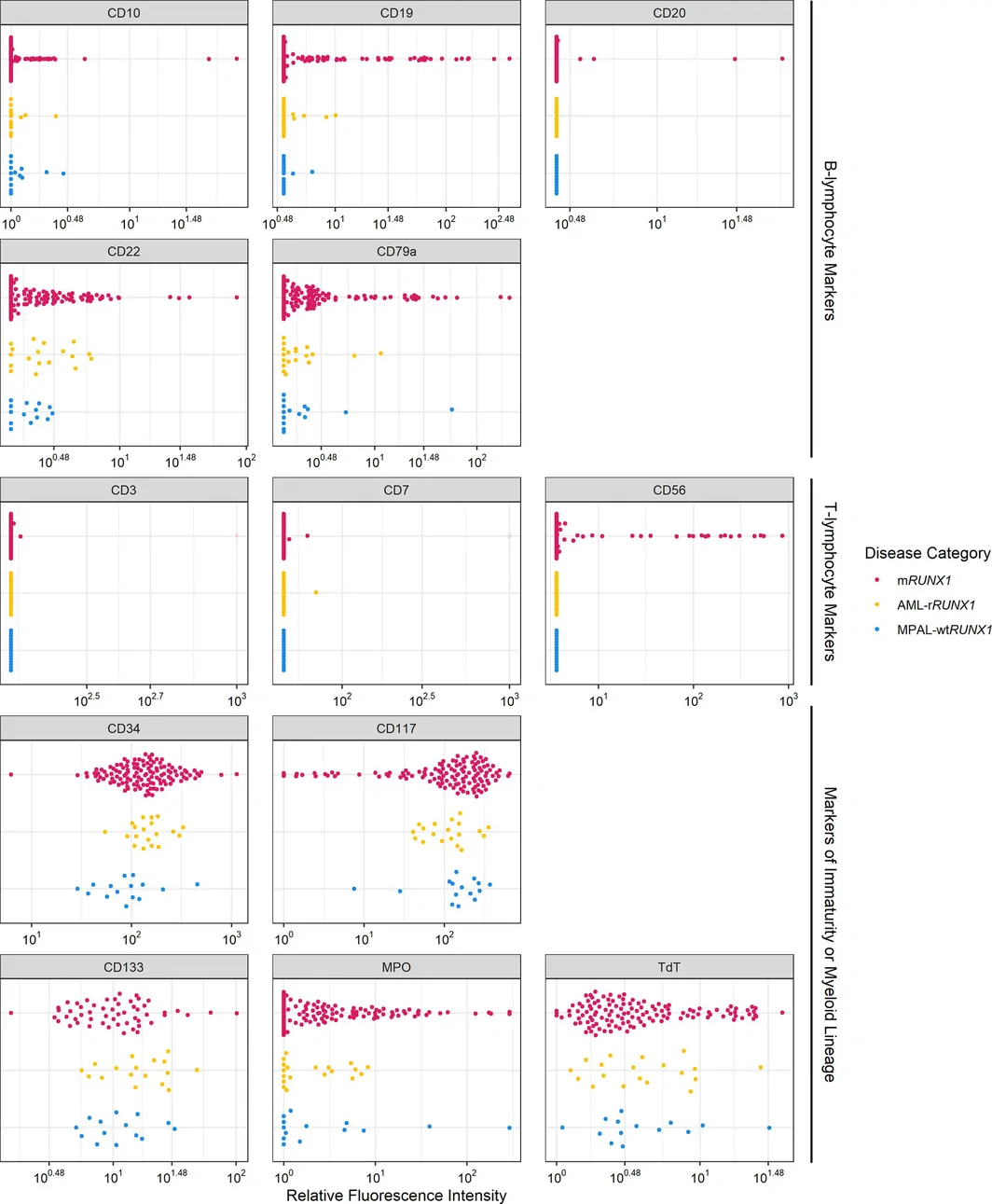
The marker expression did not appear grossly different between the diagnostic groups qualitatively. However, pairwise comparisons found CD34 had increased expression in the m*RUNX1* and AML‐r*RUNX1* groups versus the MPAL‐wt*RUNX1* group, CD19 had increased expression in the m*RUNX1* group versus the MPAL‐wt*RUNX1* group, and CD56 had increased expression in the m*RUNX1* group versus the MPAL‐wt*RUNX1* and AML‐r*RUNX1* groups. Figure S1. Relative fluorescence intensity distribution of the m*RUNX1* with variants of functional significance, AML‐r*RUNX1*, and MPAL‐wt*RUNX1* groups. Figure S2. Relative fluorescence intensity distribution of the m*RUNX1* with variants of unclear significance, AML‐r*RUNX1*, and MPAL‐wt*RUNX1* groups. Figure S3. Relative fluorescence intensity distribution of the m*RUNX1* with truncating mutations, AML‐r*RUNX1*, and MPAL‐wt*RUNX1* groups. Figure S4. Relative fluorescence intensity distribution of the m*RUNX1* with nontruncating mutations, AML‐r*RUNX1*, and MPAL‐wt*RUNX1* groups.

Evaluation of blasts for recurring immunophenotypic aberrancies in a panel of antigens commonly assessed at diagnosis (CD10, CD19, CD20, CD22, CD79a, CD7, CD56, CD3, MPO, CD117, CD34, CD133, and TdT) demonstrated no overt differences in antigen expression patterns for blasts in the AL‐m*RUNX1* and MDS‐m*RUNX1* groups, and therefore subsequent analyses were performed using a combined m*RUNX1* group. Qualitatively, the phenotypic profiles for marker expression did not appear grossly different between the m*RUNX1* group and the comparator groups AML‐r*RUNX1* and MPAL‐wt*RUNX1* that are known to be associated with characteristic phenotypic aberrancies (Figure 3) [13]. Similarly, no clear qualitative differences were observed in subgroups comprised of individuals with variants of known functional significance (Figure S1) and variants of uncertain significance (Figure S2), and between truncating (Figure S3) and nontruncating *RUNX1* mutations (Figure S4). The comparison of marker expression as quantified by RFI between diagnostic groups using the Kruskal‐Wallis test identified no significant association. However, exploratory pairwise comparisons using Dunn's test found increased expression of CD34, CD19, and CD56 in the m*RUNX1* group compared to the MPAL‐wt*RUNX1* group (CD34: Adj. *p* = 0.01941; CD19: Adj. *p* = 0.03726; CD56: Adj. *p* = 0.04422). At least three B‐lymphoid markers demonstrated positivity (> 80% blasts above negative control MFI) in 14.4%, 31.6%, and 33.3% (18/125; 6/19; 5/15) of patients in the m*RUNX1*, AML‐r*RUNX1*, and MPAL‐wt*RUNX1* groups, respectively. Within MPAL‐wt*RUNX1*, B/myeloid and B/T subtypes both had 50% of cases (2/4; 1/2) have at least three positive B‐lymphoid markers.

The Kruskal–Wallis test did not demonstrate any significant associations in marker expression based on *RUNX1* mutation region. Exploratory pairwise comparisons using Dunn's test found significant differences in CD10 expression, with increased overall expression in cases with *RUNX1* mutations in the RHD compared to the undefined region in the AL‐m*RUNX1* group (Adj. *p* = 0.02764), and similar trends overall (Adj. *p* = 0.06101). The Kruskal‐Wallis test also did not find any significant association in marker expression in patients with different *RUNX1* mutation types. Exploratory pairwise comparisons using Dunn's test found significant differences in CD79a expression, with greater expression in patients with missense mutations compared to truncating mutations overall (Adj. *p* = 0.004452) and in the AL‐m*RUNX1* group (Adj. *p* = 0.01584).

## 4 Discussion

This retrospective study aimed to characterize the immunophenotype, genetics, and clinical characteristics of patients with *RUNX1* mutations in acute and chronic myeloid neoplasms. Through this, we sought to identify associations between clinical, pathologic, or genetic features and immunophenotypic aberrancies that may complicate diagnosis of myeloid neoplasms carrying these mutations.

Evaluation of patients with myeloid neoplasms harboring *RUNX1* mutations in this cohort showed that missense *RUNX1* mutations were enriched in the RHD, a region with mutations associated with disruptions in DNA binding and loss‐of‐function [4, 9, 20]. Frameshift mutations appear to cluster in the C‐terminus, which has also been previously observed (Figure 1) [21]. Next, thirteen previous hotspot mutations in the RHD have been identified, of which our data set contains cases that include eight of those (R107, R134, R162, R166, S167, D198, R201, and R204) [22]. The comparative frequencies within these hotspot mutations have not been well described in previous literature, although R166, R201, and R204 were some of the earliest described and among the more frequently observed hotspot mutations in AML with m*RUNX1*, which our data corroborate [23, 24]. The substantial proportion of *RUNX1* mutations (42.1%) with a high VAF (> 40%) appears consistent with a recent study that reported about 30% frequency in germline *RUNX1* mutations in an AML cohort, but this comparison would require further evaluation of germline status to be definitive [25]. Lastly, analysis of outcomes within subgroups based on mutation characteristics yielded few significant associations, likely attributable to smaller sample sizes. However, one notable finding was that platelet count at diagnosis for patients with *RUNX1* mutations in the undefined region was significantly greater than the RHD and TAD. This may be due to the presence of germline mutations associated with *RUNX1* familial platelet disorder that have been commonly seen in the RHD and TAD [8], although this would also require confirmation of germline status of the mutations in our data.

The most common comutations observed in patients with *RUNX1* mutations were *ASXL1*, *SRSF2*, and *TET2* for both AL‐m*RUNX1* and MDS‐m*RUNX1* (Figure 2). *ASXL1* and *TET2* are known to be common mutations in AML, and *ASXL1* mutations have been previously observed to commonly cooccur with *RUNX1* [26, 27]. For *SRSF2*, previous studies have shown mixed results regarding its frequency as a *RUNX1* comutation compared to other genes [24, 28, 29]. *ASXL1* and *SRSF1* comutations have previously been shown to confer an inferior prognosis, with AML cases that contain these mutations at diagnosis being classified in the adverse risk category of the 2022 European LeukemiaNet (ELN) recommendations [24, 30]. The impact of *TET2* on prognosis in AML is less clear, in which most previously studied cohorts demonstrated no significant difference in outcome [31].

While the overall immunophenotypic expression amongst the m*RUNX1*, AML‐r*RUNX1*, and MPAL‐wt*RUNX1* groups did not appear grossly different (Figure 3), the reported similarities and differences by pairwise analysis in the expression of CD19 may have further diagnostic implications. As such, these differences may aid in understanding the spectrum of phenotypic aberrancy in acute leukemias with *RUNX1* mutations and their similarities and differences from bona fide MPAL, in which expression of CD19 is a key diagnostic criterion [15, 16]. Notably, CD19 can be used as a target for chimeric antigen receptor (CAR) T‐cell therapy or other immunotherapies such as blinatumomab, which have demonstrated response in AML‐r*RUNX1* and B/myeloid MPAL [32, 33], and CD19‐directed CAR T‐cell therapy has also been reported by Ma et al. to effectively target cytogenetically normal CD19‐positive AML blast cells in vitro [34]. While not current standard of practice, targeted therapy exploiting aberrant expression of CD19 by myeloid blasts may warrant future exploration [35]. Although the precise mechanism of aberrant B‐lineage antigen expression associated with changes in *RUNX1* is not fully understood, evidence suggests possible roles of PAX5 activation by MAP kinase and the myeloid master regulator transcription factor PU.1 in AML‐r*RUNX1* [36, 37]. While our study did not specifically examine mechanisms underlying aberrant B‐lineage antigen expression, the increased CD10 expression associated with *RUNX1* mutations in the RHD and increased CD79a expression associated with *RUNX1* missense mutations that were observed to cluster in the RHD potentially suggests a mechanism for aberrancy that may involve interactions with DNA. This may be a distinct mechanism compared to mutations in non‐DNA‐binding domains that often truncate the protein [21], but possibly retain *RUNX1* function as it pertains to marker expression.

Key limitations of our study include the variable availability of clinical and immunophenotypic data and the lack of standard internal flow cytometry (isotype) controls due to the retrospective nature of our project, which only included data from routine clinical testing that does not regularly incorporate such controls (however, the application of internal control cell populations facilitates a degree of standardization to account for the latter). Inconsistent availability of flow cytometry data for patients with *RUNX1* mutations and limited evaluation of potential germline variants in the cohort, both of which result from reliance on standard clinical care pathways, in which such testing is not routinely performed in all patients, partially limits the scope of this descriptive study. However, as the primary provincial referral laboratory for acute leukemia flow cytometry, it is likely that the spectrum of phenotypic aberrancy in the cohort with acute leukemia, in which flow cytometry is most often indicated, is relatively well captured. Subtle or heterogeneous aberrancy may be better studied by utilizing unsupervised clustering and machine learning tools to identify recurrent patterns of immunophenotypic aberrancy not evident in manual review, sub‐stratifying diseases that currently appear homogeneous into distinct entities based on phenotypic parameters. Moreover, the delineation of positive from negative expression is inherently arbitrary and challenging to uniformly apply across heterogeneous populations. A number of somatic abnormalities associated with lineage infidelity are included in updated classification systems applicable to acute leukemia; further exploration of the association between somatic mutations and cross‐lineage immunophenotypic aberrancy appears to be warranted to improve the pathologic definition of entities currently defined phenotypically and facilitate their objective risk stratification.

In this cohort, we observed myeloid neoplasms with *RUNX1* mutations to show B‐lineage immunophenotypic aberrancy similar to that present in AML‐r*RUNX1* and MPAL with B‐lineage involvement analyzed using identical flow cytometry panels [15, 30]. We also observed a substantial proportion of higher‐risk comutations that may contribute to the adverse risk associations of *RUNX1* mutations. Further evaluation of associations of specific patterns of immunophenotypic aberrancy and somatic mutations in large cohorts of patients with leukemia is warranted and may enable meaningful separation of the currently heterogenous and phenotypically defined acute leukemias of ambiguous lineage.

## Author Contributions

Y.H.X. contributed to study design, chart review, data analysis and visualization, and manuscript preparation. E.M. contributed to study design, research ethics applications, and manuscript preparation.

## Funding

Funding was received from the University of British Columbia Faculty of Medicine Summer Student Research Program (Award #5728).

## Ethics Statement

This study was approved by the BC Cancer Research Ethics Board (H23‐02261). No identifying patient information is included in this article.

## Consent

Patient consent for this retrospective chart review was waived by the BC Cancer Research Ethics Board.

## Conflicts of Interest

The authors declare no conflicts of interest.

## Supporting information


**Figure S1:** Relative fluorescence intensity distribution of the m*RUNX1* with variants of functional significance, AML‐r*RUNX1*, and MPAL‐wt*RUNX1* groups.
**Figure S2:** Relative fluorescence intensity distribution of the m*RUNX1* with variants of unclear significance, AML‐r*RUNX1*, and MPAL‐wt*RUNX1* groups.
**Figure S3:** Relative fluorescence intensity distribution of the m*RUNX1* with truncating mutations, AML‐r*RUNX1*, and MPAL‐wt*RUNX1* groups.
**Figure S4:** Relative fluorescence intensity distribution of the m*RUNX1* with nontruncating mutations, AML‐r*RUNX1*, and MPAL‐wt*RUNX1* groups.

## Data Availability

The data that support the findings of this study are available on request from the corresponding author. The data are not publicly available due to privacy or ethical restrictions.

## References

[1] R. Sood , Y. Kamikubo , and P. Liu , “Role of RUNX1 in Hematological Malignancies,” Blood 129, no. 15 (2017): 2070–2082, 10.1182/blood-2016-10-687830. 28179279 PMC5391618

[2] G. Swiers , M. De Bruijn , and N. A. Speck , “Hematopoietic Stem Cell Emergence in the Conceptus and the Role of Runx1,” International Journal of Developmental Biology 54, no. 6–7 (2010): 1151–1163, 10.1387/ijdb.103106gs. 20711992 PMC4512753

[3] N. A. Speck and D. G. Gilliland , “Core‐Binding Factors in Haematopoiesis and Leukaemia,” Nature Reviews. Cancer 2, no. 7 (2002): 502–513, 10.1038/nrc840. 12094236

[4] L. Zhang , S. M. Lukasik , N. A. Speck , and J. H. Bushweller , “Structural and Functional Characterization of Runx1, CBFβ, and CBFβ‐SMMHC,” Blood Cells, Molecules & Diseases 30, no. 2 (2003): 147–156, 10.1016/S1079-9796(03)00022-6. 12732176

[5] H. Miyoshi , K. Shimizu , T. Kozu , N. Maseki , Y. Kaneko , and M. Ohki , “T(8;21) Breakpoints on Chromosome 21 in Acute Myeloid Leukemia Are Clustered Within a Limited Region of a Single Gene, AML1,” Proceedings of the National Academy of Sciences 88, no. 23 (1991): 10431–10434, 10.1073/pnas.88.23.10431. PMC52942 1720541

[6] M. Osato , “Point Mutations in the RUNX1/AML1 Gene: Another Actor in RUNX Leukemia,” Oncogene 23, no. 24 (2004): 4284–4296, 10.1038/sj.onc.1207779. 15156185

[7] C. L. Wan , Y. H. Huang , S. M. Huang , et al., “Investigations of the Prognostic Value of RUNX1 Mutation in Acute Myeloid Leukemia Patients: Data From a Real‐World Study,” Leukemia Research 139 (2024): 107483, 10.1016/j.leukres.2024.107483. 38493755

[8] C. C. Homan , H. S. Scott , and A. L. Brown , “Hereditary Platelet Disorders Associated With Germ Line Variants in *RUNX1*, *ETV6*, and *ANKRD26* ,” Blood 141, no. 13 (2023): 1533–1543, 10.1182/blood.2022017735. 36626254 PMC10651873

[9] P. A. Greif , N. P. Konstandin , K. H. Metzeler , et al., “RUNX1 Mutations in Cytogenetically Normal Acute Myeloid Leukemia Are Associated With a Poor Prognosis and Up‐Regulation of Lymphoid Genes,” Haematologica 97, no. 12 (2012): 1909–1915, 10.3324/haematol.2012.064667. 22689681 PMC3590097

[10] T. Haferlach , A. Stengel , S. Eckstein , et al., “The New Provisional WHO Entity ‘RUNX1 Mutated AML’ Shows Specific Genetics but no Prognostic Influence of Dysplasia,” Leukemia 30, no. 10 (2016): 2109–2112, 10.1038/leu.2016.150. 27211269 PMC5056958

[11] J. D. Khoury , E. Solary , O. Abla , et al., “The 5th Edition of the World Health Organization Classification of Haematolymphoid Tumours: Myeloid and Histiocytic/Dendritic Neoplasms,” Leukemia 36, no. 7 (2022): 1703–1719, 10.1038/s41375-022-01613-1. 35732831 PMC9252913

[12] D. A. Arber , A. Orazi , R. P. Hasserjian , et al., “International Consensus Classification of Myeloid Neoplasms and Acute Leukemias: Integrating Morphologic, Clinical, and Genomic Data,” Blood 140, no. 11 (2022): 1200–1228, 10.1182/blood.2022015850. 35767897 PMC9479031

[13] K. Kita , K. Nakase , H. Miwa , et al., “Phenotypical Characteristics of Acute Myelocytic Leukemia Associated With the t(8;21)(q22;q22) Chromosomal Abnormality: Frequent Expression of Immature B‐Cell Antigen CD19 Together With Stem Cell Antigen CD34,” Blood 80, no. 2 (1992): 470–477. 1378322

[14] T. Menter , P. Lundberg , F. Wenzel , et al., “ ** *RUNX1* ** Mutations Can Lead to Aberrant Expression of CD79a and PAX5 in Acute Myelogenous Leukemias: A Potential Diagnostic Pitfall,” Pathobiology 86, no. 2–3 (2019): 162–166, 10.1159/000493688. 30396184

[15] G. V. George , M. Kajstura , A. N. Jajosky , et al., “Association Between B‐Cell Marker Expression and RUNX1 Lesions in Acute Myeloid Leukemia, Beyond RUNX1::RUNX1T1 Fusion: Diagnostic Pitfalls With Mixed‐Phenotype Acute Leukemia—B/Myeloid,” Cancers 17, no. 8 (2025): 1354, 10.3390/cancers17081354. 40282530 PMC12026294

[16] B. S. George , B. Yohannan , A. Gonzalez , and A. Rios , “Mixed‐Phenotype Acute Leukemia: Clinical Diagnosis and Therapeutic Strategies,” Biomedicine 10, no. 8 (2022): 1974, 10.3390/biomedicines10081974. PMC9405901 36009521

[17] R Core Team , “R: A Language and Environment for Statistical Computing,” R Foundation for Statistical Computing, 2024, https://www.R‐project.org/.

[18] H. Wickham , M. Averick , J. Bryan , et al., “Welcome to the Tidyverse,” Journal of Open Source Software 4, no. 43 (2019): 1686, 10.21105/joss.01686.

[19] T. M. Therneau and P. M. Grambsch , Modeling Survival Data: Extending the Cox Model (Springer, 2001).

[20] A. E. Quesada , G. Montalban‐Bravo , R. Luthra , et al., “Clinico‐Pathologic Characteristics and Outcomes of the World Health Organization (WHO) Provisional Entity de Novo Acute Myeloid Leukemia With Mutated RUNX1,” Modern Pathology 33, no. 9 (2020): 1678–1689, 10.1038/s41379-020-0531-2. 32238878

[21] N. D. Jayne , Z. Liang , D. H. Lim , et al., “RUNX1 C‐Terminal Mutations Impair Blood Cell Differentiation by Perturbing Specific Enhancer‐Promoter Networks,” Blood Advances 8, no. 10 (2024): 2410–2423, 10.1182/bloodadvances.2023011484. 38513139 PMC11112616

[22] X. Luo , S. Feurstein , S. Mohan , et al., “ClinGen Myeloid Malignancy Variant Curation Expert Panel Recommendations for Germline RUNX1 Variants,” Blood Advances 3, no. 20 (2019): 2962–2979, 10.1182/bloodadvances.2019000644. 31648317 PMC6849945

[23] W. J. Song , M. G. Sullivan , R. D. Legare , et al., “Haploinsufficiency of CBFA2 Causes Familial Thrombocytopenia With Propensity to Develop Acute Myelogenous Leukaemia,” Nature Genetics 23, no. 2 (1999): 166–175, 10.1038/13793. 10508512

[24] V. I. Gaidzik , V. Teleanu , E. Papaemmanuil , et al., “RUNX1 Mutations in Acute Myeloid Leukemia Are Associated With Distinct Clinico‐Pathologic and Genetic Features,” Leukemia 30, no. 11 (2016): 2160–2168, 10.1038/leu.2016.126. 27137476

[25] L. Simon , J. F. Spinella , C. Y. Yao , et al., “High Frequency of Germline *RUNX1* Mutations in Patients With *RUNX1* ‐Mutated AML,” Blood 135, no. 21 (2020): 1882–1886, 10.1182/blood.2019003357. 32315381

[26] D. R. Richardson , D. M. Swoboda , D. T. Moore , et al., “Genomic Characteristics and Prognostic Significance of Co‐Mutated * asxl1/srsf2 * Acute Myeloid Leukemia,” American Journal of Hematology 96, no. 4 (2021): 462–470, 10.1002/ajh.26110. 33502020 PMC10284351

[27] M. Khan , J. Cortes , T. Kadia , et al., “Clinical Outcomes and Co‐Occurring Mutations in Patients With RUNX1‐Mutated Acute Myeloid Leukemia,” International Journal of Molecular Sciences 18, no. 8 (2017): 1618, 10.3390/ijms18081618. 28933735 PMC5578010

[28] K. Wang , F. Zhou , X. Cai , H. Chao , R. Zhang , and S. Chen , “Mutational Landscape of Patients With Acute Myeloid Leukemia or Myelodysplastic Syndromes in the Context of RUNX1 Mutation,” Hematology 25, no. 1 (2020): 211–218, 10.1080/16078454.2020.1765561. 32476595

[29] R. C. Lindsley , B. G. Mar , E. Mazzola , et al., “Acute Myeloid Leukemia Ontogeny Is Defined by Distinct Somatic Mutations,” Blood 125, no. 9 (2015): 1367–1376, 10.1182/blood-2014-11-610543. 25550361 PMC4342352

[30] H. Döhner , A. H. Wei , F. R. Appelbaum , et al., “Diagnosis and Management of AML in Adults: 2022 Recommendations From an International Expert Panel on Behalf of the ELN,” Blood 140, no. 12 (2022): 1345–1377, 10.1182/blood.2022016867. 35797463

[31] Q. Gao , K. Shen , and M. Xiao , “TET2 Mutation in Acute Myeloid Leukemia: Biology, Clinical Significance, and Therapeutic Insights,” Clinical Epigenetics 16, no. 1 (2024): 155, 10.1186/s13148-024-01771-2. 39521964 PMC11550532

[32] A. Plesa , H. Labussière‐Wallet , S. Hayette , G. Salles , X. Thomas , and P. Sujobert , “Efficiency of Blinatumomab in a t(8;21) Acute Myeloid Leukemia Expressing CD19,” Haematologica 104, no. 10 (2019): e487–e488, 10.3324/haematol.2019.225557. 31197067 PMC6886432

[33] L. Wang , Y. Pang , C. Fang , et al., “Favorable Response of a Patient With Primary B/Myeloid Mixed Phenotype Acute Leukemia to CD19‐CAR‐T: Case Report and Literature Review,” Medicine (Baltimore) 102, no. 50 (2023): e36397, 10.1097/MD.0000000000036397. 38115347 PMC10727594

[34] G. Ma , Y. Wang , T. Ahmed , et al., “Anti‐CD19 Chimeric Antigen Receptor Targeting of CD19 + Acute Myeloid Leukemia,” Leukemia Research Reports 9 (2018): 42–44, 10.1016/j.lrr.2018.03.002. 29892548 PMC5993359

[35] O. Wolach and R. M. Stone , “How I Treat Mixed‐Phenotype Acute Leukemia,” Blood 125, no. 16 (2015): 2477–2485, 10.1182/blood-2014-10-551465. 25605373

[36] D. Ray , S. Y. Kwon , H. Tagoh , O. Heidenreich , A. Ptasinska , and C. Bonifer , “Lineage‐Inappropriate PAX5 Expression in t(8;21) Acute Myeloid Leukemia Requires Signaling‐Mediated Abrogation of Polycomb Repression,” Blood 122, no. 5 (2013): 759–769, 10.1182/blood-2013-02-482497. 23616623

[37] S. K. Zaidi , C. R. Dowdy , A. J. Van Wijnen , et al., “Altered Runx1 Subnuclear Targeting Enhances Myeloid Cell Proliferation and Blocks Differentiation by Activating a miR‐24/MKP‐7/MAPK Network,” Cancer Research 69, no. 21 (2009): 8249–8255, 10.1158/0008-5472.CAN-09-1567. 19826043 PMC2995702

