# Supplementary material for: Immunophenotypic, Genetic, and Clinical Features Associated With RUNX1 Mutation in Acute Leukemias and Chronic Myeloid Neoplasms
Source: Int J Lab Hematol. 2026 May 15;48(5):1094–102. doi: 10.1111/ijlh.70145 (PMC13555192; doi:10.1111/ijlh.70145)
Supplement: Supplementary file 1 [file IJLH-48-1094-s001.docx]

Figure S1. Relative fluorescence intensity distribution of the m*RUNX1* with variants of functional significance, AML-r*RUNX1*, and MPAL-wt*RUNX1* groups.

 Figure S2. Relative fluorescence intensity distribution of the m*RUNX1* with variants of unclear significance, AML-r*RUNX1*, and MPAL-wt*RUNX1* groups.

 Figure S3. Relative fluorescence intensity distribution of the m*RUNX1* with truncating mutations, AML-r*RUNX1*, and MPAL-wt*RUNX1* groups.

 Figure S4. Relative fluorescence intensity distribution of the m*RUNX1* with non-truncating mutations, AML-r*RUNX1*, and MPAL-wt*RUNX1* groups.
